# Supplementary material for: Machine Learning Accelerates Crystallization for Structure Determination
Source: Angew Chem Int Ed Engl. 2026 May 4;65(25):e1218503. doi: 10.1002/anie.1218503 (PMC13266958; doi:10.1002/anie.1218503)

checkCIF/PLATON report

Structure factors have been supplied for datablock(s) lcz041

THIS REPORT IS FOR GUIDANCE ONLY. IF USED AS PART OF A REVIEW PROCEDURE FOR PUBLICATION, IT SHOULD NOT REPLACE THE EXPERTISE OF AN EXPERIENCED CRYSTALLOGRAPHIC REFEREE.

No syntax errors found. CIF dictionary Interpreting this report

Datablock: lcz041

Bond precision:	C-C = 0.0077 A		Wavelength=1.54184
Cell:	a=12.7396(2)	b=12.0638(1)	c=24.1304(2)
	alpha=90	beta=90.057(1)	gamma=90
Temperature:	100 K		

	Calculated	Reported
Volume	3708.55(7)	3708.55(7)
Space group	P 21/n	P 1 21/n 1
Hall group	-P 2yn	-P 2yn
Moiety formula	C31 H17 Ag3 F18 N6 O2	C15 H3 Ag3 F18 N6, C16 H14 O2
Sum formula	C31 H17 Ag3 F18 N6 O2	C31 H17 Ag3 F18 N6 O2
Mr	1171.12	1171.11
Dx,g cm-3	2.098	2.098
Z	4	4
Mu (mm-1)	13.807	13.807
F000	2256.0	2256.0
F000'	2266.61	
h,k,lmax	16,15,30	15,15,30
Nref	7988	7436
Tmin,Tmax	0.143,0.166	0.719,1.000
Tmin'	0.064	

Correction method= # Reported T Limits: Tmin=0.719 Tmax=1.000
AbsCorr = MULTI-SCAN

Data completeness= 0.931 Theta(max)= 78.506

R(reflections)= 0.0415(6391) wR2(reflections)= 0.1147(7436)

S = 1.128 Npar= 542

The following ALERTS were generated. Each ALERT has the format
test-name_ALERT_alert-type_alert-level.
Click on the hyperlinks for more details of the test.

Alert level C

```
PLAT029_ALERT_3_C _diffrn_measured_fraction_theta_full value Low .      0.973 Why?
PLAT042_ALERT_1_C Calc. and Reported MoietyFormula Strings  Differ     Please Check
              Calc: C31 H17 Ag3 F18 N6 O2
              Rep.: C15 H3 Ag3 F18 N6, C16 H14 O2
PLAT213_ALERT_2_C Atom F01F            has ADP max/min Ratio .....        3.1 prolat
PLAT213_ALERT_2_C Atom F01G            has ADP max/min Ratio .....        3.7 prolat
PLAT220_ALERT_2_C NonSolvent   Resd 1  F   Ueq(max)/Ueq(min) Range        3.4 Ratio
PLAT911_ALERT_3_C Missing FCF Refl Between Thmin & STh/L=    0.600        184 Report
               -1  0  1,   1  0  1,  11  0  1,   8  0  2,  15  0  3,  15  1  3,
                8  0  4,  15  1  4,  15  0  5,  15  1  5,  14  0  6,  14  1  6,
               14  3  6,  14  1  7,  14  2  7,  14  3  7,  14  4  7,  14  0  8,
               13  1  8,  14  1  8,  14  2  8,  13  3  8,  14  3  8,  14  4  8,
               12  8  8,  13  0  9,  13  1  9,  14  1  9,  11  2  9,  13  2  9,
                   (  154 More Missing: see the .ckf listing file)
```

Alert level G

```
PLAT083_ALERT_2_G SHELXL Second Parameter in WGHT  Unusually Large       9.68 Why ?
PLAT142_ALERT_4_G s.u. on b - Axis Small or Missing .............    0.00010 Ang.
PLAT143_ALERT_4_G s.u. on c - Axis Small or Missing .............    0.00020 Ang.
PLAT242_ALERT_2_G Low    'MainMol' Ueq as Compared to Neighbors of       C016 Check
PLAT242_ALERT_2_G Low    'MainMol' Ueq as Compared to Neighbors of       C01E Check
PLAT242_ALERT_2_G Low    'MainMol' Ueq as Compared to Neighbors of       C01H Check
PLAT242_ALERT_2_G Low    'MainMol' Ueq as Compared to Neighbors of       C01J Check
PLAT242_ALERT_2_G Low    'MainMol' Ueq as Compared to Neighbors of       C01K Check
PLAT242_ALERT_2_G Low    'MainMol' Ueq as Compared to Neighbors of       C01M Check
PLAT434_ALERT_2_G Short Inter HL..HL Contact F00L     ..F00R     .       2.84 Ang.
                                         3/2-x,-1/2+y,1/2-z  =      2_645 Check
PLAT720_ALERT_4_G Number of Unusual/Non-Standard Labels ..........         77 Note
              Ag01    Ag02    Ag03    F004    F005    F006    F007    F008
              O009    F00A    F00B    F00C    N00D    N00E    F00F    N00G
              N00H    N00I    F00J    N00K    F00L    O00M    F00N    F00O
              F00P    F00Q    F00R    C00S    C00T    C00U    C00V    H00V
              C00W    H00W    C00X    C00Y    C00Z    H00Z    C010    H010
              C011    C012    C013    C014    H014    C015    C016    C017
              H017    C018    C019    H019    C01A    H01A    C01B    H01B
              C01C    H01C    C01D    H01D    C01E    F01F    F01G    C01H
              C01I    H01I    C01J    C01K    C01L    H01L    C01M    C01N
              H01N    C01O    H01E    H01F    H01G
PLAT883_ALERT_1_G Absent Datum for _atom_sites_solution_primary ..     Please Do !
PLAT912_ALERT_4_G Missing # of FCF Reflections Above STh/L=  0.600        349 Note
PLAT941_ALERT_3_G Average HKL Measurement Multiplicity ..........         3.9 Low
```

```
PLAT969_ALERT_5_G The 'Henn et al.' R-Factor-gap value ..........      2.606 Note
              Predicted wR2: Based on SigI**2  4.40 or SHELX Weight 10.17
PLAT978_ALERT_2_G Number C-C Bonds with Positive Residual Density.         0 Info
```

```
   0 ALERT level A = Most likely a serious problem - resolve or explain
   0 ALERT level B = A potentially serious problem, consider carefully
   6 ALERT level C = Check. Ensure it is not caused by an omission or oversight
  16 ALERT level G = General information/check it is not something unexpected

   2 ALERT type 1 CIF construction/syntax error, inconsistent or missing data
  12 ALERT type 2 Indicator that the structure model may be wrong or deficient
   3 ALERT type 3 Indicator that the structure quality may be low
   4 ALERT type 4 Improvement, methodology, query or suggestion
   1 ALERT type 5 Informative message, check
```

Validation response form

Please find below a validation response form (VRF) that can be filled in and pasted into your CIF.

```
# start Validation Reply Form
_vrf_PLAT029_lcz041
;
PROBLEM: _diffrn_measured_fraction_theta_full value Low .      0.973 Why?
RESPONSE: ...
;
_vrf_PLAT042_lcz041
;
PROBLEM: Calc. and Reported MoietyFormula Strings  Differ     Please Check
RESPONSE: ...
;
_vrf_PLAT213_lcz041
;
PROBLEM: Atom F01F            has ADP max/min Ratio .....        3.1 prolat
RESPONSE: ...
;
_vrf_PLAT220_lcz041
;
PROBLEM: NonSolvent   Resd 1  F   Ueq(max)/Ueq(min) Range        3.4 Ratio
RESPONSE: ...
;
_vrf_PLAT911_lcz041
;
PROBLEM: Missing FCF Refl Between Thmin & STh/L=    0.600        184 Report
RESPONSE: ...
;
# end Validation Reply Form
```

It is advisable to attempt to resolve as many as possible of the alerts in all categories. Often the minor alerts point to easily fixed oversights, errors and omissions in your CIF or refinement strategy, so attention to these fine details can be worthwhile. It is up to the individual to critically assess their own results and, if necessary, seek expert advice.

PLATON version of 17/09/2025; check.def file version of 17/09/2025

duplicate check

No duplication found

Datablock lcz041 - ellipsoid plot

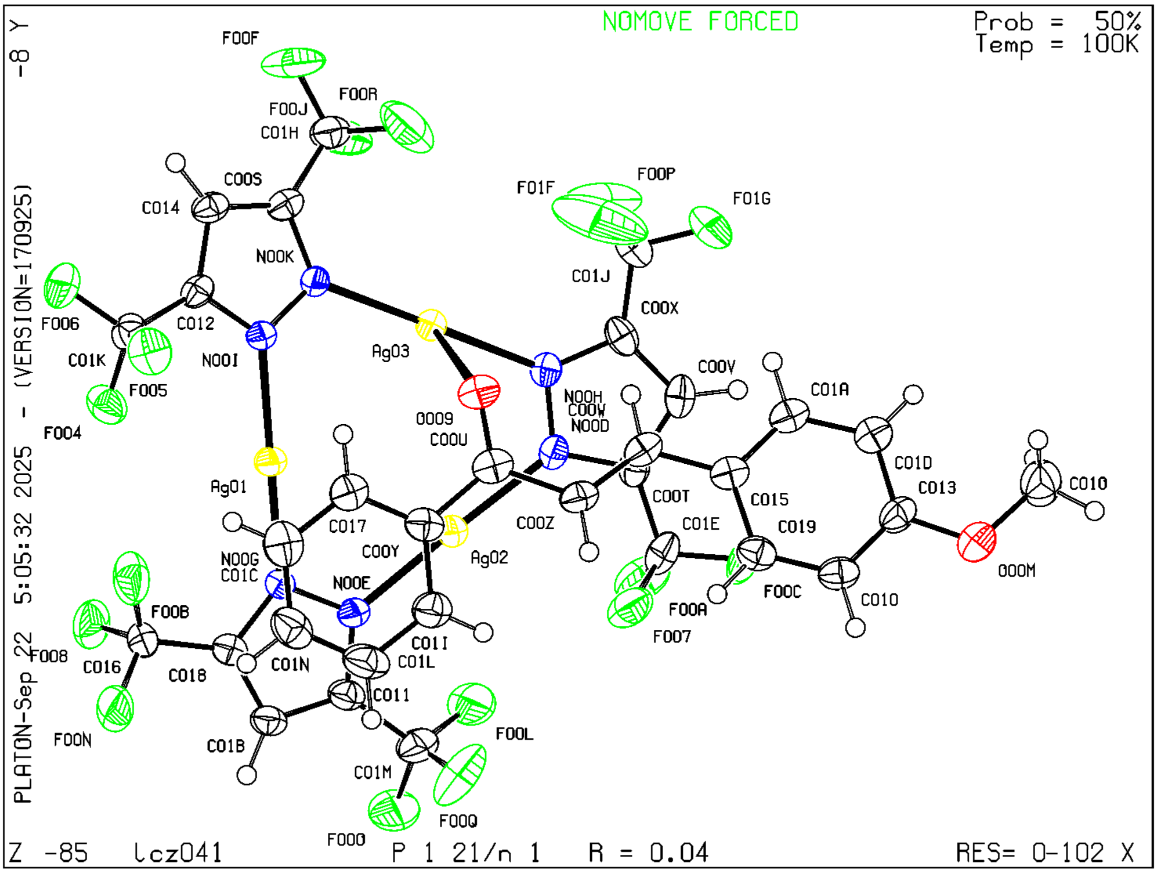

Supplement: Supplementary file 2 — Supporting File 2: anie72462‐sup‐0002‐DataFile.zip. [file ANIE-65-e1218503-s002.zip › supplementary_materials_crystallographic_data submit.zip!/cif_and_checkcif(1-114)/Ag3Pz3_41/Ag3Pz3_41_checkcif.pdf]
